# The influence of age at symptom onset and length of followup on mortality in patients with recent-onset inflammatory polyarthritis

**DOI:** 10.1002/art.23402

**Published:** 2008-04

**Authors:** Sophia M Naz, Tracey M Farragher, Diane K Bunn, Deborah P M Symmons, Ian N Bruce

**Affiliations:** 1 University of ManchesterManchester, UK and Chesterfield and North Derbyshire Royal Hospital NHS TrustChesterfield, UK; 2 University of ManchesterManchester, UK; 3 Norfolk and Norwich University HospitalNorwich, UK

## Abstract

**Objective:**

To investigate the influence of age at symptom onset and length of followup on mortality in patients with recent-onset inflammatory polyarthritis (IP), and to examine predictors of mortality in relation to disease duration.

**Methods:**

From 1990 to 1994, patients with recent-onset IP were registered with the Norfolk Arthritis Register (NOAR) and followed up prospectively. Standardized mortality ratios (SMRs) were calculated for all-cause and cardiovascular disease (CVD) mortality and for those who were younger than age 55 years at disease onset and for the first 5 and 10 years of followup. Cox proportional hazards models were developed to assess predictors of early and later mortality.

**Results:**

Of 1,098 patients, 224 (20%) had died by the end of 2004. All-cause and CVD mortality were increased in rheumatoid factor (RF)–positive patients and in this subgroup, CVD mortality was increased at both early and later followup (SMR 5-year followup 1.93 [95% confidence interval 1.08–3.19]; SMR 10-year followup 2.00 [95% confidence interval 1.37–2.80]). CVD mortality was highest in seropositive patients <55 years of age at disease onset (SMR 5.58 [95% confidence interval 2.24–11.50]). In multivariate models, age at onset, male sex, RF positivity, Health Assessment Questionnaire score ≥1.5, and nodules were predictors of early and later mortality.

**Conclusion:**

Patients with IP had higher rates of CVD mortality throughout the followup period studied, and this was highest in seropositive patients who were <55 years of age at symptom onset. This subgroup deserves particular attention in terms of disease and risk factor modification. Nodules were independent predictors of CVD mortality, suggesting that extraarticular/vascular inflammation identifies patients at particularly high CVD risk.

Rheumatoid arthritis (RA) is associated with premature mortality. The excess mortality risk is highest in patients with established disease duration of >5 years (1). Three recent studies of early RA showed no increase in mortality (2–4). Cardiovascular disease (CVD) mortality is particularly increased in RA (5–8) and there is evidence that excess CVD mortality begins early, especially in rheumatoid factor (RF)–positive patients (5–8). Solomon et al also recently reported that the highest relative risk of CVD mortality was in young RA patients (<50 years of age), while the highest absolute excess risk was in those >75 years of age (9). However, they did not examine all-cause mortality or the specific effect of age at onset.

Using the Norfolk Arthritis Register (NOAR), we tested the hypotheses that age at onset of IP and disease duration influence all-cause and CVD mortality. We also compared the predictors of mortality in early versus more established disease.

## PATIENTS AND METHODS

### The Norfolk Arthritis Register

Briefly, the NOAR is a primary care–based inception cohort of patients with early IP. The registry covers the area of the former Norwich Health Authority, with a population of ∼500,000. Primary care providers notified the NOAR of patients who were age 16 years or older at symptom onset, had swelling of at least 2 joints that had persisted for at least 4 weeks, and had symptom onset after January 1, 1989. A parallel notification system operated from hospitals within the catchment area (7,10). Between 1990 and 1994, 1,098 patients with recent-onset IP were registered with the NOAR.

### Baseline data

At baseline, a research nurse performed a structured interview and clinical examination. Demographic and clinical parameters, including the number of swollen and tender joints (range 0–51), duration of morning stiffness, and the presence of rheumatoid nodules, were recorded. A blood sample was obtained for RF testing (latex agglutination technique). RF was considered positive at a titer of ≥1:40. Patients completed a Health Assessment Questionnaire (HAQ), modified for use in British patients (11). Patients were required to meet the 1987 American College of Rheumatology (ACR; formerly, the American Rheumatism Association) criteria for RA (12).

### Notification and cause of death

All patients were flagged with the Office for National Statistics (ONS) and followed up from the date of symptom onset to December 31, 2004. Patients who left the UK (n = 4; 0.36%) were censored on the date of “embarkation.” Causes of death were coded by the ONS from death certificate data, using the International Classification of Diseases, Ninth Revision (ICD-9) until December 31, 2000, and the International Statistical Classification of Diseases and Related Health Problems, Tenth Revision (ICD-10) from January 1, 2001 onward (13). For this study, deaths were attributed to CVD if CVD (chapter 7 in ICD-9 or chapter I in ICD-10) was mentioned anywhere on the death certificate.

### Statistical analysis

#### Standardized mortality ratios (SMRs)

Age-and sex-specific mortality rates in the Norfolk population for the years 1990–2003 were provided by the ONS and were used to calculate the expected numbers of deaths in the NOAR cohort. Observed and expected numbers of deaths (all-cause and CVD, specifically) were compared using SMRs (with 95% confidence intervals [95% CIs]), which were calculated for all patients with IP, then separately for men and women. We also stratified patients according to whether they satisfied the ACR 1987 classification criteria for RA at baseline, and according to RF status at baseline.

We first examined mortality rates for patients who were ≤55 years of age at IP symptom onset and those who were ≤65 years of age at onset of IP. We also examined mortality rates for both the first 5 years of followup and the first 10 years of followup.

#### Predictors of mortality

Potential baseline predictors of mortality for the whole cohort were initially assessed univariately using Cox proportional hazards regression. Baseline predictor variables that were significant at *P* ≤ 0.20 on univariate analysis were entered into a multivariate model, which was simplified using the likelihood ratio test. Variables that were significant at *P* < 0.10 on multivariate analysis were retained. Previously excluded variables (*P* ≤ 0.20) were added back into the model and those that were significant at *P* < 0.10 were retained. The proportional hazards assumption of the final multivariate model was checked (7). All statistical analyses were conducted with Stata software, release 9 (StataCorp, College Station, TX).

## RESULTS

We studied 1,098 patients, of whom 715 (65%) were women. The median age at symptom onset was 54 years (interquartile range [IQR] 41–67) and the median delay from symptom onset to registration was 5 months (IQR 2–10). At baseline, 499 patients (45%) met the ACR criteria for RA. RF was measured in 958 patients, of whom 267 (27.9%) were RF positive. The median followup time was 11.4 years (IQR 10–12.75). By December 31, 2004, 224 patients (20%; 115 women and 109 men) had died. CVD was the most common cause of death (46 women [40%] and 46 men [42%]). Cancer was the second most common cause of death (28 women [24%] and 36 men [33%]).

In the entire cohort, all-cause mortality was not increased (SMR 1.06 [95% CI 0.93–1.21]), but there was a significant increase in CVD mortality (SMR 1.25 [95% CI 1.01–1.54]) (Table 1). All-cause and CVD mortality were not increased in those who satisfied the ACR criteria for RA at baseline or in those with a longer length of followup (Table 1)

**Table 1:** SMRs in patients with inflammatory polyarthritis, by sex, length of followup, RA and RF status, and age at symptom onset*

	All-cause mortality			CVD mortality		
		
	Observed	Expected	SMR (95% CI)	Observed	Expected	SMR (95% CI)
Sex						
Both sexes	224	211.3	1.06 (0.93–1.21)	92	73.4	1.25 (1.01–1.54)
Female	115	117.5	0.98 (0.81–1.17)	46	39.3	1.16 (0.86–1.56)
Male	109	94.0	1.16 (0.95–1.40)	46	34.1	1.35 (0.99–1.80)
Length of followup						
5 years	86	91.3	0.94 (0.75–1.16)	35	29.5	1.19 (0.83–1.65)
10 years	187	186.9	1.00 (0.86–1.15)	77	64.1	1.20 (0.95–1.50)
Satisfied ACR criteria for RA at baseline						
Yes	123	113.1	1.09 (0.90–1.30)	44	37.8	1.17 (0.85–1.56)
No	101	98.4	1.03 (0.84–1.25)	48	35.7	1.34 (0.99–1.78)
RF status at baseline						
Positive	78	55.7	1.40 (1.11–1.75)	38	19.0	2.00 (1.41–2.74)
Negative	114	129.6	0.88 (0.73–1.06)	43	46.6	0.92 (0.67–1.24)
RF status at baseline: findings at 5-year followup						
Positive	33	23.8	1.39 (0.96–1.95)	15	7.8	1.93 (1.08–3.19)
Negative	42	53.3	0.79 (0.57–1.07)	16	17.8	0.90 (0.51–1.46)
RF status at baseline: findings at 10-year followup						
Positive	63	49.2	1.28 (0.98–1.64)	33	16.5	2.00 (1.37–2.80)
Negative	94	113.4	0.83 (0.67–1.01)	33	40.4	0.82 (0.56–1.15)
Age at symptom onset						
Entire cohort						
<55 years	20	18.9	1.06 (0.64–1.63)	9	5.1	1.75 (0.80–3.32)
≥55 years	204	192.6	1.06 (0.92–1.22)	83	68.3	1.21 (0.97–1.51)
RF-positive patients						
<55 years	11	4.38	2.51 (1.25–4.48)	7	1.3	5.58 (2.24–11.50)
≥55 years	67	51.4	1.30 (1.01–1.66)	31	17.7	1.75 (1.19–2.48)

* SMRs = standardized mortality ratios; RA = rheumatoid arthritis; RF = rheumatoid factor; 95% CI = 95% confidence interval; CVD = cardiovascular disease; ACR = American College of Rheumatology.

All-cause mortality was significantly increased in RF-positive patients (SMR 1.40 [95% CI 1.11–1.75]), as was CVD mortality (SMR 2.00 [95% CI 1.41–2.74]). This excess CVD mortality was already apparent 5 years after symptom onset and persisted through 10 years of followup (Table 1). In RF-positive patients, the risk of death from all causes, and especially from CVD, was higher in those younger than age 55 years at symptom onset (SMR for death from all causes 2.51 [95% CI 1.25–4.48]; SMR for CVD mortality 5.58 [95% CI 2.24–11.50]). The number of excess deaths (observed deaths minus expected deaths) was higher for RF-positive patients age 55 years or older at symptom onset, both for all causes (15.6 deaths for age ≥55 years and 6.6 for age <55 years) and for CVD (13.3 for age ≥55 years and 5.7 for age <55 years). RF-positive women younger than age 55 years at disease onset had the highest SMR for CVD (6.13 [95% CI 1.26–17.92]).

### Predictors of mortality

In univariate analysis, age at symptom onset and sex were significant predictors of all-cause and CVD mortality at both early and later followup (Table 2). Patients who presented late (>8 months from symptom onset) were less likely to die during followup than those who presented earlier (hazard ratio [HR] 0.56 [95% CI 0.34–0.93]). There was an unexpected relationship between smoking and mortality. Compared with never smokers, ex-smokers who stopped ≥15 years prior to baseline had a higher mortality risk (HR 2.06 [95% CI 1.16–3.65]). This was not seen for ex-smokers who stopped <15 years prior to registration (HR 1.54 [95% CI 0.85–2.79]). Nodules and RF positivity were both strong predictors of early CVD mortality (HR 2.16 [95% CI 0.92–5.07] and HR 2.41 [95% CI 1.34–4.34], respectively) and later CVD mortality (HR 1.95 [95% CI 1.05–3.64] and HR 2.43 [95% CI 1.61–3.67], respectively). The presence of nodules also predicted early and later all-cause mortality.

**Table 2:** Baseline variables and predictors of all-cause and CVD mortality, by length of followup*

		HR (95%CI)			
		
		5-year followup		10-year followup	
			
Variable	Entire IP cohort (n=1.098)	All-cause mortality	CVD mortality	All-cause mortality	CVD mortality
Age at symptom onset (10-year intervals), median (IQR)	54 (41–67)	2.73 (2.26–3.28)	3.50 (2.64–4.64)	2.59 (2.29–2.95)	2.87 (2.39–3.45)
Male, no. (%)	383 (35)	2.81 (1.84–4.28)	2.30 (1.32–3.98)	2.16 (1.62–2.87)	2.25 (1.54–3.29)
Smoking status, no. (%)					
Never smoked	353 (32)	1.0	1.0	1.0	1.0
Ex-smokers who stopped ≥15 years prior to registration	209 (19.0)	2.06 (1.16–3.65)	2.40 (1.13–5.08)	1.40 (0.94–2.09)	1.62 (0.94–2.77)
Ex-smokers who stopped <15 years prior to registration	237 (21.6)	1.54 (0.85–2.79)	1.41 (0.62–3.19)	1.13 (0.76–1.70)	1.31 (0.76–2.26)
Current smokers	290 (26.4)	1.19 (0.65–2.16)	1.44 (0.52–3.97)	1.14 (0.77–1.51)	1.41 (0.76–2.62)
Delay to presentation in months (tertiles), no. (%)					
Group 1 (0–2.9 months)	366 (33.3)	1.00	1.00	1.00	1.00
Group 2 (3.0–7.9 months)	368 (33.5)	0.50 (0.30–0.85)	0.33 (0.16–0.72)	0.87 (0.62–1.21)	0.73 (0.46–1.14)
Group 3 (8.0–54.0 months)	364 (33.1)	0.56 (0.34–0.93)	0.57 (0.30–1.07)	0.63 (0.44–0.92)	0.60 (0.37–0.97)
RF positive at baseline, no. (%)	267 (27.9)	2.09 (1.34–3.27)	2.41 (1.34–4.34)	1.85 (1.35–2.53)	2.43 (1.61–3.67)
Nodules present at baseline, no. (%)	73 (6.6)	2.70 (1.53–4.78)	2.16 (0.92–5.07)	2.19 (1.43–3.37)	1.95 (1.05–3.64)
Satisfied ACR criteria at baseline, no. (%)	499 (45)	1.00 (0.66–1.52)	0.68 (0.38–1.21)	1.36 (1.02–1.80)	1.30 (0.89–1.91)
HAQ score at baseline, no. (%)					
<1.5	832 (76.6)	1.00	1.00	1.00	1.00
≥1.5	254 (23.4)	1.80 (1.39–2.34)	1.94 (1.30–2.91)	1.80 (1.50–2.16)	1.70 (1.33–2.18)

* IP = inflammatory polyarthritis; HR = hazard ratio; IQR = interquartile range; HAQ = Health Assessment Questionnaire (see Table 1 for other definitions).

In multivariate analysis, older age at symptom onset, male sex, and RF positivity were consistent predictors of all-cause and CVD mortality in both followup periods (Table 3). A high baseline HAQ score also predicted later all-cause mortality. Interestingly, nodules remained a significant predictor of mortality (all-cause, HR 2.12 [95% CI 1.33–3.37] and CVD, HR 2.28 [95% CI 1.17–4.46]), independent of RF status.

**Table 3:** Multivariate predictors of all-cause and CVD mortality, by length of followup*

	HR (95% CI)			
	
	5-year followup		10-year followup	
		
Variable	All-cause mortality	CVD mortality	All-cause mortality	CVD mortality
Age at symptom onset (10-year intervals)	2.71 (2.17–3.39)	3.89 (2.77–5.46)	2.50 (2.15–2.91)	2.94 (2.37–4.46)
Sex	2.28 (1.44–3.60)	1.64 (0.89–3.00)	2.19 (1.59–3.02)	2.08 (1.38–3.16)
Delay to presentation in months (tertiles)				
Group 1 (0–2.9 months)	1.00	†	†	†
Group 2 (3.0–7.9 months)	0.52 (0.30–0.92)	†	†	†
Group 3 (8.0–54.0 months)	0.92 (0.53–1.60)	†	†	†
RF-positive at baseline	1.59 (1.01–2.51)	2.95 (1.58–5.50)	1.45 (1.01–2.05)	1.99 (1.31–3.01)
Nodules present at baseline	2.19 (1.18–4.05)	2.46 (0.91–6.71)	2.12 (1.33–3.37)	2.28 (1.17–4.46)
HAQ score at baseline ≥1.5	1.17 (0.87–1.60)	†	1.29 (1.03–1.62)	†

* HR = hazard ratio; HAQ = Health Assessment Questionnaire (see Table 1 for other definitions).

† Baseline variable subsequently shown to not be a predictor in the multivariate model.

## DISCUSSION

Using this primary care–based early IP cohort, we found that excess all-cause and CVD mortality was confined to patients who were RF positive at baseline. Excess CVD mortality was already apparent within 5 years of symptom onset, and the highest SMR for CVD deaths was in RF-positive women younger than age 55 years at symptom onset (SMR 6.13 [95% CI 1.26–17.92]).

The low number of deaths in patients who were younger at symptom onset precluded further analysis. We found that patients older than age 55 years at symptom onset had a higher rate of excess all-cause and CVD deaths. This is consistent with the observations of Solomon et al (9). Numerically, therefore, in an RA population the majority of CVD-related deaths will still be seen in those patients who were older at symptom onset. While one would expect the SMR to drop with increasing length of followup, in this study CVD mortality in the RF-positive cohort remained high, even at 10 years of followup. This suggests that exposure to chronic inflammation throughout the disease course carries an excess risk of CVD death.

The 2 strongest independent disease-associated predictors of CVD mortality were RF status and the presence of nodules. Interestingly, nodules remained in our models independent of the effect of RF status. RF-positive patients tend to have more progressive and severe disease with reduced physical function, all of which may contribute to increased mortality at younger ages, particularly from CVD. In addition, the presence of nodules may reflect a different stage in RA pathogenesis. Patients with nodules generally have more severe extraarticular manifestations, and this inflammation in the “vascular” compartment may accelerate atherogenesis further and contribute to plaque instability and a higher event rate. Patients with extraarticular disease in general may also have even fewer functional reserves to survive a significant CV event. For example, Wolfe et al (14) found the HAQ score to be the most significant predictor of mortality, and found that laboratory, radiographic, and physical examination data were substantially weaker in predicting mortality. We found that a HAQ score of ≥1.5 was an independent predictor of mortality from all causes at 10 years of followup. This could be because it is a marker of cumulative inflammation and joint damage. Earlier in the disease course, baseline markers of disease severity, such as RF status and the presence of nodules, may be better indicators of the degree of inflammation and disability.

Smoking was the only traditional CV risk factor examined in this cohort. It was not found to be a significant predictor of mortality. However, there were trends toward associations of past smoking with mortality outcomes. In the context of IP, smoking may influence mortality via its effect on RF status and nodule formation. In this cohort, 35% of the RF-positive individuals were smokers and 41% were ex-smokers.

Strengths of this study include the complete followup, via the ONS, of a large primary care–based inception cohort over a substantial time period. It is the first study to examine the impact of age at disease onset on both all-cause and CVD mortality. Although basing the cause of death on death certificate data may lead to misclassification, the degree of misclassification is likely to be similar in both the IP and the general population, and therefore would not bias the mortality risk. The main limitation of this study is that we were unable to examine the influence of other classic CVD risk factors.

In conclusion, in the NOAR cohort we found that excess all-cause and CVD mortality was confined to RF-positive patients. Excess CVD mortality was already apparent within 5 years of symptom onset, and the highest SMR for CVD death was seen in RF-positive women who were younger than age 55 years at symptom onset. However, older patients had a higher number of excess deaths from CVD. Among our IP patients, older age at symptom onset, male sex, RF positivity, and presence of nodules all predicted CVD mortality. Extraarticular/vascular inflammation may contribute to CVD mortality over and above the influence of RF status alone. These results suggest that primary CVD prevention and aggressive management of inflammation are important from the time of presentation with IP in patients of all ages.

## AUTHOR CONTRIBUTIONS

Dr. Bruce had full access to all of the data in the study and takes responsibility for the integrity of the data and the accuracy of the data analysis.

**Study design.** Symmons, Bruce.

**Acquisition of data.** Bunn, Symmons, Bruce.

**Analysis and interpretation of data.** Naz, Farragher, Symmons, Bruce.

**Manuscript preparation.** Naz, Farragher, Symmons, Bruce.

**Statistical analysis.** Naz, Farragher.
